# The patient experience of pulmonary hypertension: a large cross-sectional study of UK patients

**DOI:** 10.1186/s12890-019-0827-5

**Published:** 2019-03-21

**Authors:** Iain Armstrong, Catherine Billings, David G. Kiely, Janelle Yorke, Carl Harries, Shaun Clayton, Wendy Gin-Sing

**Affiliations:** 1 Sheffield Pulmonary Vascular Disease Unit, Royal Hallamshire Hospital, Sheffield, S10 2JF UK; 2 Pulmonary Hypertension Association UK (PHA-UK), Chapeltown, Sheffield, UK; 3 Department of Infection, Immunity and Cardiovascular Disease, University of Sheffield, Sheffield, UK; 4 School of Health Sciences, University of Manchester, Oxford Road, Manchester, M139PL UK; 5 Pulmonary Hypertension Unit, Royal Brompton and Harefield NHS Foundation Trust, London, UK; 6 Pulmonary Hypertension CNS, Hammersmith Hospital, Du Cane Road, London, W12 0HS UK

**Keywords:** Pulmonary hypertension, Real-life research, PHA-UK, Patient survey

## Abstract

**Background:**

Pulmonary Hypertension Association UK (PHA-UK) is the only charity in the UK especially for people affected by pulmonary hypertension (PH). To better understand the impact of PH on patients and carers beyond clinical symptoms, the PHA-UK carried out a cross-sectional survey on the effect of PH on daily living, along with a follow-up survey assessing the financial impact of PH.

**Methods:**

This is a descriptive cross-sectional survey of adult patients with PH in the UK. A quantitative survey of four key topics (time to diagnosis, quality of life [QoL], financial impact and specialist treatment), was made available to PHA-UK members and patients on PH therapy, with a follow-up financial impact survey sent to those responders who agreed to be contacted further. Data collection was carried out in January and February 2017 for the main survey, and November and December 2017 for the financial impact survey.

**Results:**

The main survey was completed by 567 individuals, and the financial follow-up survey by 171. Mean age of responders was 69 ± 17 years with 70% female. 60% of respondents said PH had a major impact on their QoL, with 45% reporting that treatment and management improves their QoL ‘a lot’. The time between first experiencing symptoms and diagnosis was ≥1 year for 48% of patients, with 40% seeing 4+ doctors before diagnosis. 63% of patients reported financial worries. Patients in part-time and full-time work reported the greatest financial burden, with a 13 and 33% fall in monthly income respectively. Patients had positive experiences of treatment in specialist centres, with 62% rating their care ‘excellent’, and 92% saying they preferred travelling to a specialist centre rather than seeing a local non-specialist.

**Conclusions:**

This study reports the largest UK survey exploring issues affecting patients with PH. The study shows that despite the availability of new therapies, patients are still experiencing delays prior to diagnosis, and experiencing both emotional and financial impacts from the disease. By identifying the areas patients find most important in their treatment, this research can inform future care policies and long-term management to support patients living with PH and their families.

**Electronic supplementary material:**

The online version of this article (10.1186/s12890-019-0827-5) contains supplementary material, which is available to authorized users.

## Background

Pulmonary hypertension (PH) is a pathophysiological disorder of high blood pressure, defined by a resting mean pulmonary artery pressure (mPAP) of ≥25 mmHg, measured through right heart catheterisation [1]. Patients with PH may experience a range of chronic symptoms, including dyspnoea, fatigue, cough, dizziness or syncope, chest pain, cardiac arrhythmias, oedema of the ankles and legs, ascites, or heart failure. One form of PH, pulmonary arterial hypertension (PAH), causes a progressive increase in pulmonary vascular resistance, leading ultimately to right ventricular failure and death. Estimates from large-scale national database studies suggest the global prevalence of any cause of PH is about 1% of the population, increasing to 10% in people over 65 years old [2]. In France, a national registry of 17 university hospitals in October 2002 showed that PAH was most likely to be detected late in the course of the disease [3]. Similarly, the REVEAL Registry (Registry to EValuate Early And Long-term pulmonary arterial hypertension disease management) in the USA in 2006 [4] found that over 20% of patients waited more than 2 years from the onset of symptoms attributable to PAH and diagnosis [5]. An Australian database of adults from four tertiary referral centres with idiopathic PAH (IPAH) between January 2007 and December 2008 found an even longer delay of 3.9 years [6]. Other registries used for PH research are the Giessen PH registry in Germany [7] and the ASPIRE (Assessing the Spectrum of Pulmonary hypertension Identified at a REferral centre) registry in the UK [8], which have both been used to explore factors linked with improved survival in patients with PH.

While large-scale database studies capture information about medical treatment and management of the disease in a diverse patient population, they often do not capture the patient response to treatment, or unmet needs in the day-to-day life of patients and their carers. In 2013, a large international survey of 326 patients with PAH and 129 carers from five different European countries was carried out to provide an insight into experiences of living with PH beyond clinical symptoms [9]. The survey, consisting of both qualitative one-to-one interviews and a quantitative opinion-based survey questionnaire, explored four areas affected by PAH (physical and practical, emotional, social and information needs). The results showed that PAH had a significant physical and practical impact on the daily life of both patients and their carers, and was associated with a significant emotional and financial burden. PAH also had a considerable impact on patients’ intimacy, relationships, emotional and social well-being [9]. A national survey of 114 Chinese patients with PAH using semi-structured, face-to-face interviews revealed both physical, emotional and financial burdens, along with social isolation caused by a lack of public understanding of the disease [10].

Despite advances in our understanding of PH and how it impacts patients, and despite the advent of new therapies for PAH and chronic thromboembolic pulmonary hypertension (CTEPH) [2, 3], PH remains a debilitating illness that has a major impact on the lives of patients and their carers. In order to improve patient care and disease management, it is important to gain an even greater understanding of the impact of PH and PH treatment from the patient perspective, and to identify unmet needs in the patient journey.

In the UK, patients with PAH and CTEPH (who have the disease as a direct consequence of changes in the pulmonary vasculature) are most commonly treated in nine specialist centres across the country [5]. The Pulmonary Hypertension Association UK (PHA-UK) is the only charity in the UK that advocates exclusively for people affected by PH. To gain information on how PH affects daily living, PHA-UK conducted surveys in 2007, 2010 and 2017. Here we report the results of the 2017 survey along with a follow-up survey assessing the financial impact of PH. The aim was to gather updated information on patients’ views concerning PH treatment and management. This report should provide a vital patient perspective for informing policy regarding the treatment and management of individuals with PH, and to advocate for the interests of people with PH in future debates concerning policies and treatment review. The survey provides an important platform to allow patients to share their experiences and highlight issues that are of most importance to them.

## Methods

This was a descriptive, cross-sectional survey of adult patients with PH in the UK. Data were collected via a questionnaire designed to focus on the patient experience of the disease, which explored four key topics: time to diagnosis, quality of life (QoL), financial impact and experience of specialist treatment. A further questionnaire evaluating financial impact of PH in greater depth was sent out to all those who had agreed to be contacted for further surveys. Ethical approval was not requested for the study, as no National Health Service (NHS) or patient data were collected outside of the survey responses. A contact telephone number was provided for patients if they had any further questions about the survey or data, and both the cover letter for the main survey and the questionnaire for the financial survey explained that all patient information would be kept anonymous and confidential. Consent was implied by virtue of completing and returning the form, and therefore no additional informed consent form was provided.

The questionnaire was modified from previous PAH-UK questionnaires put together by a panel of specialist physicians and reviewed by patient representatives and patient advocacy groups. All questions were designed to be patient-centric and focus on the everyday experiences of patients in order to capture the issues that were most important to the patient regarding treatment and specialist care. New questions not posed in the previous questionnaires were agreed by internal discussion between the authors, and reviewed by a patient panel to ensure that they were relevant and sufficiently comprehensive to capture the patient experience. The questionnaire sent to patients was a quantitative survey consisting of 38 separate questions grouped into five sections; ‘before diagnosis’, ‘diagnosis’, ‘treatment’, ‘living with pulmonary hypertension’ and ‘about you’ (see Additional files 1 and 2). Other than ‘current age’ and ‘age at diagnosis’ all questions were worded as closed questions with discrete pre-coded response options (e.g. ‘agree/disagree’ or ‘major impact/some impact/no impact’). There was also space for patients to write in any additional thoughts or feelings about the impact of the disease on their life.

Data collection was carried out from January 2017 to February 2017 for the main survey and November 2017 to December 2017 for the financial impact follow-up survey. The main survey was distributed via the PHA-UK charity magazine, online link and specialist centres and via the home care delivery network. A printed hard copy of the questionnaire was sent to all members of PHA-UK, along with an explanatory cover letter and pre-addressed envelope for returning the questionnaire. In order to widen inclusion to non PHA-UK members, envelopes containing the survey and cover letter were distributed via a home care delivery network to patients on PH-specific targeted therapy.

The follow-up financial impact questionnaire was a quantitative survey asking 31 separate questions divided into six sections; ‘about you’, ‘before your diagnosis’, ‘after your diagnosis’, ‘costs related to healthcare’, ‘insurance costs’ and ‘general costs’. Questions asking for numerical values (quantities of money or number of healthcare visits) were open, with a space for respondents to write in values. All other questions were closed with discrete pre-coded response options (see Additional files 1 and 2). Patients who completed the questionnaire were offered the option of entering a draw to win £10 shopping vouchers. The financial impact questionnaire was sent by post to all individuals completing the main questionnaire who agreed to be contacted further. Fifteen individuals who responded to the financial impact questionnaire were chosen at random for a qualitative telephone interview with a PH specialist, with the interview carried out with the first eight who responded. All interviews were recorded and transcribed, and informed consent was taken at the start of the call.

Forms completed online were inputted directly into the analysis spreadsheet, while data from the paper forms were checked for accurate data entry then inputted into the analysis spreadsheet by hand by the statistician. When all data were entered, a selection of questionnaires were checked for accuracy against the paper versions. Summary statistics were used to describe individuals’ responses to the questionnaire as agreed a priori by all authors, and the data were analysed using IBM SPSS v22.

## Results

Questionnaires were sent out to all members of the PHA-UK via the charity magazine, specialist centres, and online link, as well as separately via the home care delivery network. A total of 567 individuals completed the main survey, with 64 responses (11.3%) from the online survey, and the rest from paper questionnaires. Four of the online responses and none of the paper questionnaires were discarded prior to data entry as they were incorrectly filled in, giving a final analysis set of 563 patients. The demographic and disease characteristics of study participants are shown in Table 1. The financial sub-analysis survey was sent to 351 individuals with responses received from 171 (48.7%). For those in the financial sub-analysis, 65% were female with a mean age of 59.5 years, similar to those responding to the main survey.

**Table 1:** Demographic and disease characteristics

	All patients (*N* = 563)
Age, mean [SD]	59 [17]
Female, n (%)	394 (70)
Age at diagnosis, mean [SD]	53 [19]
Currently on PH medication, n (%)	498 (88)
Comorbid conditions, n (%)	318 (56)
Congenital heart disease	91 (16)
Connective tissue disease	81 (14)
Portal hypertension	13 (2)
HIV infection	0 (0)
Sickle cell anaemia	0 (0)
Lung disease (e.g. COPD, pulmonary fibrosis)	56 (10)

*COPD* chronic obstructive pulmonary disease, *HIV* human immunodeficiency virus, *SD* standard deviation

### QoL

**Fig. 1:**
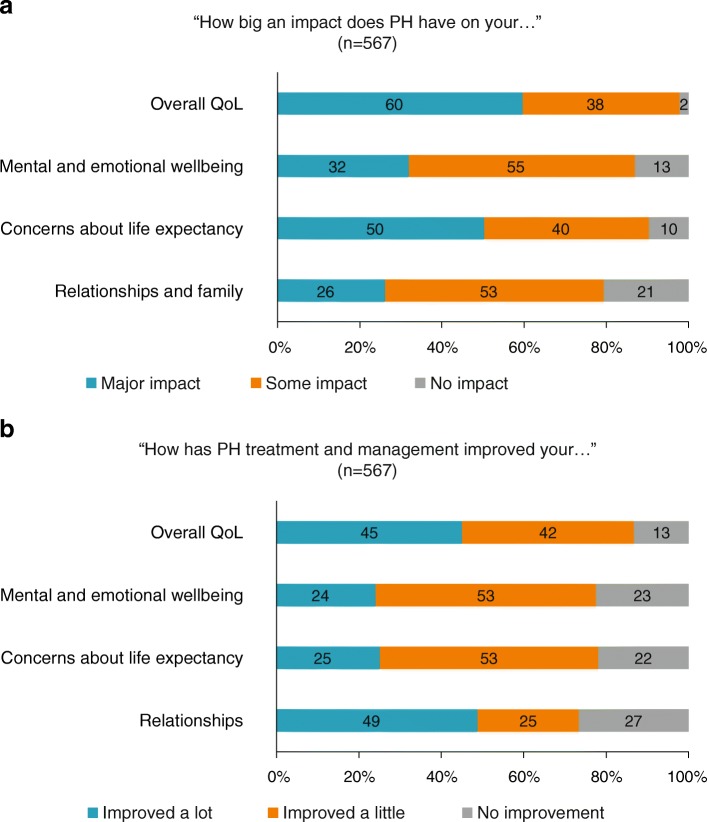
Effect of PH on QoL. (**a**) The impact of PH disease on QoL measures. (**b**) The effect of PH treatment and management on QoL measures

The results of the survey showed that 98% of patients considered their PH to have had either a ‘major impact’ or ‘some impact’ on their overall QoL. Approximately 90% reported that PH had an impact on their mental and emotional wellbeing and resulted in concerns about life expectancy. Nearly 80% of patients also reported that PH had an impact on their relationships and family (Fig. 1a).

Following treatment and management of PH, 87% of responders reported an improvement in overall QoL, with 45% reporting that their overall QoL improved ‘a lot’. The majority of patients also reported at least some improvement in their mental and emotional wellbeing following treatment and management, which also improved their concerns about life expectancy. Just under half of patients reported that their relationships improved ‘a lot’ following treatment (Fig. 1b).

When asked about their experiences of living with PH, similar percentages of patients felt they could get support to help them cope with family life and work (76%), to cope with their feelings (75%), and that they could talk about their PH-related hopes and fears (76%). Additional information added by patients indicated that PH had an impact both on physical and mental aspects of QoL, for example: “I am constantly thinking about how it has changed my life, my health, abilities and future. I am constantly worrying about what may or may not happen” (female, Cleveland). However patients were also keen to continue with their lives through treatment and management of the disease; “PH has had a huge impact on my life, however I am determined to minimise that impact as much as possible” (male, Kent).

### Experiences of diagnosis

. In the 12 months after first noticing symptoms, 30% of patients did not discuss these symptoms with a physician, and almost 48% had not received a diagnosis of PH a year after first experiencing symptoms (Table 2). A definite PH diagnosis was received by 11% of patients who saw only one HCP, while 40% of patients saw four or more HCPs and 9% saw seven or more HCPs prior to receiving a diagnosis. When asked who provided the diagnosis, 63% reported a PH centre consultant or nurse specialist, and 34% a local hospital consultant. A third of patients (33%) were admitted to hospital as an emergency case due to symptoms that resulted in a PH diagnosis. The average time to diagnosis did not differ between those diagnosed in 2015–2017 and those diagnosed pre-2015.

**Table 2:** Time to diagnosis after experiencing symptoms

	Proportion of patients, n (%)					
	< 3 months	3–6 months	6–12 months	1–2 years	2–3 years	3+ years
Time between noticing symptoms and going to see a doctor (*n* = 542)	161 (30)	128 (24)	97 (18)	68 (13)	29 (5)	59 (11)
Time between seeing general practitioner with symptoms and referral to hospital (*n* = 378)	139 (37)	87 (23)	58 (15)	40 (11)	18 (5)	35 (9)
Time before referral from local hospital to specialist pulmonary hypertension centre (*n* = 426^a^)	160 (38)	90 (21)	65 (15)	37 (9)	20 (5)	29 (7)
Time from first noticing symptoms to diagnosis with pulmonary hypertension (*n* = 545)	78 (14)	99 (18)	107 (20)	85 (16)	65 (12)	111 (20)

^a^Of these patients 25 (6%) were not referred

Patient experience of receiving their PH diagnosis showed that the majority (57%) strongly agreed that they understood their HCP’s explanation of what was wrong, whilst 27% stated they had an incomplete understanding of the disease. Responses indicated that diagnosis information was given in a sensitive manner with 65% strongly agreeing and only 5.3% strongly disagreeing. In the extra information provided, several patients reported feelings of frustration at their attempts to receive a diagnosis, or experiences of incorrect diagnoses: “I was getting more and more breathless. I kept going to my GP and they kept giving me asthma inhalers … It was upsetting and frustrating because it felt like every time I went somewhere I was told there was nothing wrong – and there clearly was.” (male, Barnsley); “It took me 8 years to get a diagnosis. I was getting increasingly breathless and I kept on going to the doctor, and kept being told it was anxiety, or I was getting unfit.” (female, Oxfordshire).

### Financial impact

Results from the main survey showed that more than half of the patients (63%) felt that PH had at least some impact on their financial situation, including their ability to attend work or education. However, for the majority of patients, treatment and management of PH neither improved their financial situation/financial worries (58%) nor their ability to attend work and education (62.7%).

Of the 171 patients in the financial sub-analysis, 35% were in full-time employment prior to diagnosis, with 14% working part time and 30% retired. Immediately after their diagnosis, of the patients who were working and responded to the question (*n* = 93), 28% were able to continue working as before, with another 28% giving up work completely, 23% on long-term sick leave and 22% reducing their hours. Table 3 shows the change in income following PH diagnosis for the 129 respondents who provided income data, based on pre-diagnosis employment status. Overall, patients in work (either full or part time) experienced large decreases in their monthly salary, while those who were retired experienced a slight increase.

**Table 3:** Percentage change in monthly income following PH diagnosis

Employment status	Mean monthly income				Change in monthly income	
	Pre-diagnosis		Post-diagnosis			
	n	£, mean (SD)	n	£, mean (SD)	n	% change
Full-time work	50	2081 (1607)	46	1386 (1238)	44	−33.3
Part-time work	21	1013 (683)	22	844 (525)	20	−13.4
Retired	39	1336 (878)	42	1385 (903)	39	1.5
Unable to work	14	897 (927)	14	773 (481)	13	0.0

*SD* standard deviation

Of the 104 patients who applied for benefits, less than half (47%) were successful at the first application, while 66% reported that the department dealing with their claim did not understand the diagnosis of PH. When asked how easy they found applying for benefits, 7% of the 104 patients reported it was ‘very easy’, 13% ‘quite easy’, 25% ‘quite difficult’, 26% ‘difficult’ and 30% ‘extremely difficult’. The patient qualitative interviews reveal stress and worry around the process of applying for benefits and appeals: “It’s a very daunting experience applying for benefits, it’s very complicated. The forms are overwhelming especially when you’re ill” (female, London); “It’s sort of like no one believes you. Like you’re lying and it’s frustrating to get across just how serious it is. Just because they don’t know about it doesn’t mean it doesn’t affect you” (female, Manchester).

In the main survey, the patient qualitative responses indicated increased costs associated with their diagnosis related to daily living and healthcare-related travel: “My electricity costs me more because I have my oxygen machine on all the time, plus a fan because the machine heats up” (female, Gateshead); “It costs me £50 in petrol to travel to my hospital appointments in London and takes nearly 3 hours. If I’m kept in, it costs my husband £55 per night to stay in hospital accommodation” (female, Portsmouth). The financial survey asked specific questions about additional costs related to healthcare, with patients reporting that since diagnosis they have spent extra money on the following: gas or electricity (53.2%), general travel costs (43.9%), help around the house or garden (43.3%), other household bills (28.7%) and, food costs or special diet food (26.3%).

### Treatment and specialist centres

The main survey found that patients generally had a very positive experience of specialist PH centres (in the UK, this is where ongoing PH treatment is prescribed and long-term care provided). The majority of patients (89%) stated that the support they received from care centres was ‘excellent’ or ‘good’. A specialist PH centre was regularly attended by 78% of respondents, with 84.6% of those stating that they felt that they saw their specialist often enough. More than 90% of respondents agreed that travelling to a specialist PH centre was preferable to being treated by non-PH specialists at a more local hospital.

Responses to questions relating to how treatment options were handled at PH specialist centres showed that 93% of patients considered that the choice of treatments was explained to them, 88% felt involved in the treatment decision, and 84% that the side-effects of treatment had been adequately explained to them. As well as providing information on medical treatment, the majority of patients reported that hospital staff provided information about support groups (68%) and the effect of PH on work and education (59%). However, fewer (40%) stated that hospital staff provided information about financial support. The disease, patient age and costs were cited by 40, 25 and 16% of patients respectively as posing the most difficulties when travelling to specialist centres.

Overall, 89.3% of patients stated that they were told who to contact for advice on coping with symptoms of side effects, with 78.6% given written information about their treatment plan. A high percentage (92.6%) reported that information regarding their disease had been given to their general practitioner, while 63% reported that such information had been given to their family or carers.

**Fig. 2:**
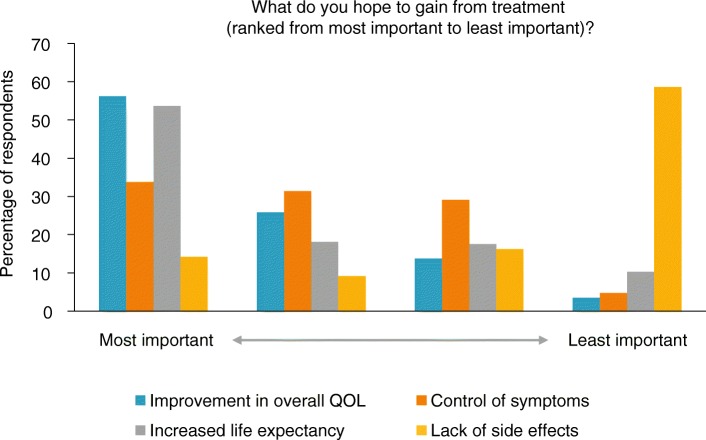
Treatment priorities

Figure 2 shows what the respondents reported hoping to gain most from their treatment. Overall, patients prioritised QoL and increased life expectancy over reducing side effects. The patient qualitative quotes often contained praise for their treatment centres and ongoing treatment support: “I can’t thank everyone at my specialist centre enough for extending my life and helping me to continue enjoying my retirement and family life” (female, Hampshire); “It’s of great comfort to us to know that, should we have a problem, we can pick up the phone and someone will be there” (male, Newton Stewart).

## Discussion

In this study, we report on the results of the largest UK survey exploring important issues affecting patients with PH. The results of our survey have shown that PH impacts QoL in 98% of patients. The majority of patients experience significant delays prior to diagnosis, and this problem has not improved over the previous decade. Encouragingly, however, the institution of therapy results in improvements in QoL and emotional well-being. The experience of the UK model of highly specialised services was overwhelmingly positive, consistent with previous smaller studies, and emphasises that patients are willing to travel to access specialist care. Finally, this study has quantified for the first time the very significant impact that the diagnosis of PH makes in patients in part-time and full-time work who, based on the data from those patients completing our follow-up survey, experience respective average falls in monthly income of 13 and 33% following their PH diagnosis.

Collecting patient responses, both through the survey and the qualitative answers provided by patients, is vital for understanding the full patient experience of the disease. PH is a long-term disease with an uncertain trajectory that not only affects the individual patient, but also their family. The PHA-UK questionnaire was designed to be specific to the UK health and benefit systems (where the cost of all PH medication is covered by the National Health Service), and is therefore able to capture the issues and worries specific to the UK PH population. Determining which aspects of treatment and care the patient is most concerned with can inform future care policies and long-term treatment management to support patients and their families living with PH.

One concern highlighted was the lack of awareness of PH among both individuals experiencing symptoms and primary care professionals. In the PHA-UK survey almost half of patients did not receive a diagnosis until a year after first experiencing symptoms, and patient comments indicated that HCPs can sometimes be reluctant to listen to patients seeking a diagnosis and even dismissive of their concerns, with patients being given alternative diagnoses such as stress, anxiety, or asthma. The percentage of patients who received a correct diagnosis on their first approach to a HCP is low and indicative that a greater awareness of PH and its symptoms is needed, including the importance of shortness of breath as a clinical sign of more serious underlying conditions. This is supported by results from other database and survey studies which highlight that similar problems are faced worldwide by patients with PH and their families in Australia [6], France [3], and the USA [5], with delays of several years between onset of symptoms and diagnosis. As well as increasing awareness among HCPs, the PHA UK survey also indicates that increased awareness is needed in the general population to encourage those experiencing PH symptoms to approach their HCPs in a timely manner, as more than 15% of patients waited over a year before going to see a doctor with their symptoms. As PAH is a deteriorating and life-threatening condition, the sooner a patient can be diagnosed, the better for the disease outcome. Further educating patients about the process of PAH diagnosis when they first present with symptoms may also help them to appreciate the importance of reporting symptoms as soon as possible, and help them through the diagnosis process.

While patients in the PHA-UK survey generally reported that treatment and management improved their QoL with PH, there was less of an improvement reported in mental, emotional and relationship issues following their treatment. Similarly, the International PAH Patient and Carer Survey in Europe [9] reported a large emotional burden of PAH on both patients and carers, with an impact on both social and emotional wellbeing, and high levels of mental health conditions such as anxiety and depression have been reported in patients with PH [11, 12]. The emotional burden was also highlighted in the study from China, along with concerns of isolation and loneliness [10]. These non-physical issues are often difficult to capture in database studies or clinical trials, yet they are vitally important for fully understanding the patient experience of the disease, and developing fully comprehensive care. The development of comprehensive care for patients should include an awareness of all of these issues, and an understanding of the emotional and informational needs of patients.

The additional financial survey allowed a greater exploration of the financial impact of PH. The results are supported by similar findings from questionnaire surveys in China [10] and Europe [9], where PH had a high impact on the working ability and earning potential of patients who were working (either part time or full time) at the time of their diagnosis. It is worth remembering as well, that due to the length of time between symptoms and diagnosis, the patients were likely to have been affected by their PH in the years before their diagnosis. Many patients may therefore have already experienced a decrease in income, moved to part-time work, or taken early retirement prior to the survey. The financial questionnaire did reveal less of a financial impact on patients already in retirement, with, on average, a small increase in income reported following PH diagnosis most likely due to their eligibility for health-related benefits.

The financial survey also provided an insight into the role of the UK benefits system in providing for patients with PH, and reveals that under the current system a high proportion of patients are not receiving the necessary support. This leads to increased financial worries for patients, carers, and immediate family who may be providing financial support. The qualitative interviews carried out reveal high levels of stress and dissatisfaction with the current benefits system, with patients finding the application forms, assessments, repeated application requirements, and appeals processes a mental, emotional and physical challenge while also dealing with the medical aspects of their disease. The study also indicates a lack of awareness of PH among individuals working within the benefits system, leading to patients experiencing additional difficulties in claiming for a disease that is both rare and an ‘invisible’ illness. Similar issues with the financial, mental and emotional strain of the current benefits system have been identified through qualitative interviews of PH patients in the UK [13].

This questionnaire covers around 10% of the UK diagnosed population managed by the national PH network, and is the largest survey of PH patients in the UK carried out to date. Comparison of the demographics with the UK National Audit of PH Services indicates that it covers a representative sample of PH patients in the UK [14]. Limitations of this study are typical for this form of questionnaire-based research. In particular the sample is self-selecting for those patients willing to answer and send back the questionnaire. This is also true of the financial survey, where around half of those who agreed to be contacted further returned the questionnaire, although it is difficult to speculate how this self-selection may have affected the data. As this is a descriptive study, no further statistical analysis was carried out on associations between the data.

These data are supported by findings from other database studies and questionnaire surveys, and show the importance of considering the financial and emotional aspects of patient care, alongside medical treatment and management. They also indicate that the length of time between the first appearance of symptoms and diagnosis, due to a lack of awareness among patients and first-line HCPs, is unacceptably long in the case of this deteriorating condition. Reducing the financial hardship incurred by living with PH would also reduce the stress and emotional burden of the disease. While in the UK the cost of medication is covered by the NHS, there is still a need to increase awareness of the disease in agencies responsible for supplying benefits or financial support to individuals with PH. By engaging with patient experiences, a full understanding of the patient journey can be put together and used for public healthcare advocacy, ensuring patient needs are reflected in treatment guidelines and legislation to improve health, wellbeing and QoL of patients with PH.

## Conclusions

This study reports on the largest UK-wide survey exploring the key issues affecting patients with PH. The study shows that the time between first experiencing symptoms and final diagnosis is still too long in the case of this deteriorating condition, and that, despite the availability of new therapies, patients are experiencing both emotional and financial impacts from the disease. The study identifies the areas patients find most important in their treatment, which is important for informing future care policies and long-term management to support patients living with PH and their families.

## Additional files

Additional file 1: PHA UK survey_Appendix 1. (PDF 133 kb)

Additional file 2: PHA UK survey_Appendix 2 (PDF 92 kb)

## Abbreviations

COPD: Chronic obstructive pulmonary disease
CTEPH: Chronic thromboembolic pulmonary hypertension
HCP: Healthcare professionals
IPAH: Idiopathic pulmonary arterial hypertension
LDH: Lactate dehydrogenase
NHS: National Health Service
PAH: Pulmonary arterial hypertension
PH: Pulmonary hypertension
PHA-UK: The Pulmonary Hypertension Association UK
QoL: Quality of life
