# Supplementary material for: The patient experience of pulmonary hypertension: a large cross-sectional study of UK patients
Source: BMC Pulm Med. 2019 Mar 21;19:67. doi: 10.1186/s12890-019-0827-5 (PMC6429756; doi:10.1186/s12890-019-0827-5)

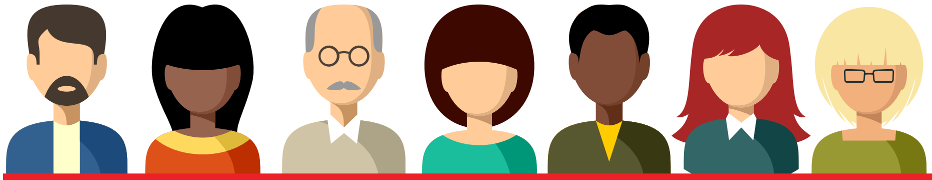

pha UK pulmonary hypertension association

Living with PH | 2016 Survey

1. Before diagnosis

The early symptoms of pulmonary hypertension can often be mild and are common to many diseases so that it is often a difficult and lengthy process to arrive at the diagnosis. We would like to ask you about your experiences.

A. *How long did you notice symptoms before going to see a doctor?*

Less than 3 mths ☐ *3-6 mnths* ☐ *6-12 mnths* ☐ *1-2yrs* ☐ *2-3yrs* ☐ *3+yrs* ☐

B. *Were you admitted to hospital as an emergency because of your symptoms which led to you being diagnosed with pulmonary hypertension?*

Yes ☐ *(go to Section 2)* *No* ☐ *(please answer the next three Questions)*

C. *If you went first to see your GP with your symptoms(and were not admitted to hospital as an emergency) how long was it before you were referred to hospital?*

Less than 3 mths ☐ *3-6 mnths* ☐ *6-12 mnths* ☐ *1-2yrs* ☐ *2-3yrs* ☐ *3+yrs* ☐

D. *If you were referred to a local hospital, how long was it before you were referred to a Specialist Pulmonary Hypertension Centre?*

Not referred ☐ *Less than 3 mnths* ☐ *3-6 mnths* ☐ *6-12 mnths* ☐ *1-2yrs* ☐ *2-3yrs* ☐ *3+yrs* ☐

E. *Which one of these Specialist Pulmonary Hypertension Centres where you referred to?*

Freeman Hospital, Newcastle ☐
Golden Jubilee National Hospital, Glasgow ☐
Great Ormond Street Hospital, London ☐
Hammersmith Hospital, London ☐
Mater Misericordiae University Hospital, Dublin ☐
Papworth Hospital NHS Trust, Cambridgeshire ☐
Royal Brompton Hospital, London ☐
Royal Hallamshire Hospital, Sheffield ☐
Royal Free Hospital, London ☐

---

2. Diagnosis

Please tell us a little more about your experience of diagnosis.

A. *Overall how long was it from the time you first noticed symptoms until you were diagnosed with pulmonary hypertension?*

Less than 3 mths ☐ *3-6 mnths* ☐ *6-12 mnths* ☐ *1-2yrs* ☐ *2-3yrs* ☐ *3+yrs* ☐

B. *How many doctors did you see before getting the diagnosis of pulmonary hypertension?*

1 ☐ *2* ☐ *3* ☐ *4* ☐ *5* ☐ *6* ☐ *7* ☐ *More than 7* ☐

C. *Who gave you your diagnosis?*

GP ☐ *Local Hospital Consultant* ☐ *Specialist PH Centre Consultant* ☐ *Other* ☐

D. ***I understood the explanation of what was wrong.***

Agree ☐ ☐ ☐ ☐ ☐ ☐ *Disagree*

E. ***I felt the information was given in a sensitive manner.***

Agree ☐ ☐ ☐ ☐ ☐ ☐ *Disagree*

3. Treatment

We'd like to hear more about how your treatment options were handled at your PH specialist centre.

A. ***The choice of treatments were explained to me.***

Agree ☐ *Disagree* ☐

B. ***I felt involved in the treatment decisions.***

Agree ☐ *Disagree* ☐

C. ***The side effects of treatments were explained to me.***

Agree ☐ *Disagree* ☐

D. ***Hospital staff gave me information about:***

Support groups	*Agree* ☐	*Disagree* ☐
Effect of PH on work and education	*Agree* ☐	*Disagree* ☐
Getting financial support	*Agree* ☐	*Disagree* ☐

E. ***I was told who to contact if I need advice on coping with symptoms or side effects.***

Agree ☐ *Disagree* ☐

F. ***I was given written information about my treatment plan.***

Agree ☐ *Disagree* ☐

G. ***Information about my care was given to my family/carers.***

Agree ☐ *Disagree* ☐

H. ***Information was given to my GP.***

Agree ☐ *Disagree* ☐

I. ***What do you most hope to gain from treatment?*** *Rank most important (1) to least important (4)*

Improvement in overall QOL ☐ *Control of symptoms* ☐

Increased life expectancy ☐ *Lack of side effects* ☐

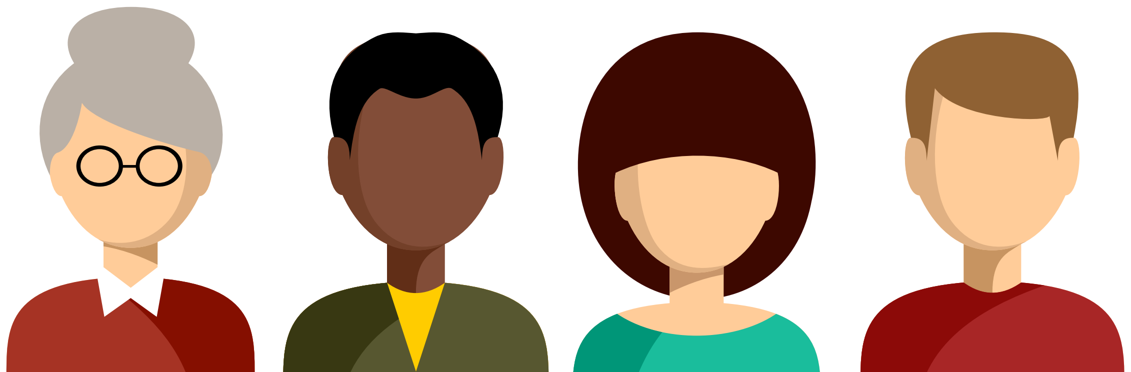

4. Living with Pulmonary Hypertension

Pulmonary hypertension can affect many areas of your life. We would like to ask you what the impact on your life has been.

A. ***How big an impact does PH have in these areas of your life?***

	Major impact	*Some impact*	*No impact*
Overall quality of life	☐	☐	☐
Concerns about life expectancy	☐	☐	☐
General mental/emotional well being	☐	☐	☐
Relationships/family	☐	☐	☐
Ability to attend work/education	☐	☐	☐
Financial situation/financial worries	☐	☐	☐

B. ***How has your PH treatment and management affected these areas of your life?***

	Improved a lot	*Improved a little*	*No Improvement*
Overall QOL	☐	☐	☐
Concerns about life expectancy	☐	☐	☐
General mental/emotional well being	☐	☐	☐
Relationships/family	☐	☐	☐
Ability to attend work/education	☐	☐	☐
Financial situation/financial worries	☐	☐	☐

C. ***Since being diagnosed how frequently do you see a pulmonary hypertension specialist doctor or nurse about your PH?***

Every month or more ☐ *Every 1-2 mnths* ☐ *Every 3-4 mnths* ☐

Every 6-12 mnths ☐ *Once a year* ☐ *Never* ☐

D. ***Do you regularly attend a Specialist Pulmonary Hypertension Centre?***

Yes ☐ *No* ☐

E. ***Do you see your specialist doctor or nurse often enough?***

Yes ☐ *No* ☐

F. ***How many miles do you have to travel to see your specialist doctor or nurse?***

0-20 miles ☐ *20-50 miles* ☐ *50-100 miles* ☐ *More than 100 miles* ☐

G. ***Do you have difficulties with travelling because of:***

Age	*Yes* ☐	*No* ☐
Condition	*Yes* ☐	*No* ☐
Cost	*Yes* ☐	*No* ☐

H. ***Do you think it is better to travel to a Specialist PH centre rather than to be under the care of a non-PH specialist at a more local hospital?***

Yes ☐ *No* ☐

I. ***How satisfied are you with the support you receive?***

Poor ☐ *Fair* ☐ *Good* ☐ *Excellent* ☐

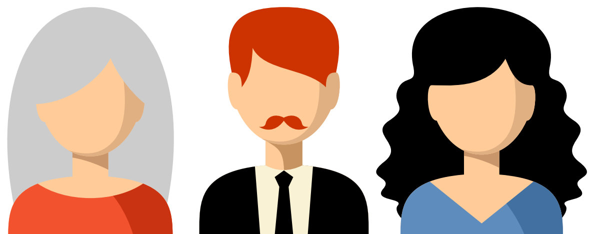

J. I feel I can get support to:

Help me cope with family life and work — Agree ☐ Disagree ☐

Help me cope with my feelings — Agree ☐ Disagree ☐

K. I feel I can talk about my hopes and fears.

Agree ☐ Disagree ☐

5. About you

We would like to know a little more about you. All information will be kept confidential.

A. What is your current age?

________ Years

B. What was your age at diagnosis?

________ Years

C. Are you:

Male ☐ Female ☐

D. Which of the following PH drugs are you currently on?

Nifedipine ☐ Diltiazem ☐ Nicardipine ☐

Amlodipine ☐ Ambrisentan (Volibris) ☐ Bosentan (Tracleer) ☐

Macitentan (Opsumit) ☐ Sildenafil (Viagra or Revatio) ☐ Tadalafil (Adcirca) ☐

Riociguat (Adempas) ☐ Epoprostenol (Flolan) ☐ Iloprost (Ventavis) ☐

Treprostinil (Remodulin) ☐ Veletri ☐

E. Have you had any of the following surgery?

Pulmonary endarterectomy ☐ Atrial septostomy ☐ Transplant surgery ☐

F. Do you have any of the following conditions?

Connective tissue disease ☐ Portal hypertension ☐ Congenital heart disease ☐

Sickle cell anaemia ☐ HIV infection (Human Immunodeficiency Virus) ☐

Lung disease (e,g, COPD, pulmonary fibrosis) ☐

G. Have you been asked to take part in research looking at new tests or drugs?

Yes ☐ No ☐

H. It is very important to ensure the patient voice continues to be heard. Would you be happy to be contacted in the future by email or mail to allow us to hear your views on topics that are of important to patients with pulmonary hypertension?

Yes ☐ No ☐ (If Yes, please provide your details)

Name ______________________

Address ______________________

Email ______________________

Thank you! We very much appreciate you taking the time to complete the ***'Living with PH 2016'*** survey. Please check you have answered all sections and then return your completed survey in the envelope provided. If you have any questions about the survey please call the PHA UK office on 01709 761450 or email office@phassociation.uk.com

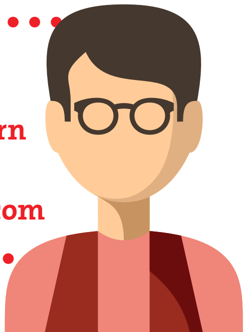

Supplement: Supplementary file 1 — PHA UK survey_Appendix 1. (PDF 133 kb) [file 12890_2019_827_MOESM1_ESM.pdf]
