# Supplementary material for: The patient experience of pulmonary hypertension: a large cross-sectional study of UK patients
Source: BMC Pulm Med. 2019 Mar 21;19:67. doi: 10.1186/s12890-019-0827-5 (PMC6429756; doi:10.1186/s12890-019-0827-5)
Supplement: Supplementary file 2 — PHA UK survey_Appendix 2 (PDF 92 kb) [file 12890_2019_827_MOESM2_ESM.pdf]

The financial impact of pulmonary hypertension

Our most recent *Living with PH* survey showed that 89% of people living with pulmonary hypertension think that the financial worries linked to their condition have an impact on their lives.

We want to know more, with the ultimate goal of helping people live better with PH.

This is the first time that research has looked specifically at the financial impact of PH, and the results of this ground-breaking study will play a vital part in helping us push for change. All responses are anonymous and confidential. Please, share your voice and help us stand up for people affected by pulmonary hypertension.

About you

We would like to know a little more about you. All information will be kept confidential.

1. ***Are you:***
   Male ☐ *Female* ☐

2. ***What is your current age?***
   ________ *Years*

3. ***What was your age at diagnosis?***
   ________ *Years*

Before your diagnosis

4. ***What was your employment situation?*** *(Mark all those that apply)*
   In full time education ☐ *Working full-time* ☐ *Working part-time* ☐ *Looking for employment* ☐
   Unable to work for medical reasons ☐ *At home with children / caring for a loved one* ☐ *Retired* ☐

5. ***Did you live:***
   Alone ☐ *With a spouse or partner* ☐ *With children under 18 years old* ☐
   With children over 18 years old ☐ *With parents* ☐ *With friends / house share* ☐

6. ***Approximately what was your monthly income?*** *(including wages after tax and deductions, and any benefits you were entitled to at the time, eg. child allowance, working tax credit, housing benefit)*
   £________________ *You* £________________ *Your partner**

**'Partner' refers to your husband, wife, or partner who lives in the same house as you.*

After your diagnosis

7. Approximately what is your monthly income now? *(including wages after tax and deductions, and any benefits you are entitled to eg. child allowance, working tax credit, housing benefit)*

£________________ You £________________ Your partner

8. Immediately after your diagnosis, how did your working situation change?

	You	Your partner/carer
Continued working as before	☐	☐
Reduced your hours	☐	☐
Taken long-term sick leave	☐	☐
Had to give up work completely	☐	☐
I was not working	☐	☐

9. What is your working situation now?

	You	Your partner/carer
I have continued working as before my diagnosis	☐	☐
I have reduced my hours	☐	☐
I have taken long-term sick leave	☐	☐
I have had to give up work completely	☐	☐
I was not working and am still not working	☐	☐

10. Have you tried to claim any benefit support since being diagnosed with PH? *(eg. DLA, PIP, ESA)*

Yes ☐ No ☐

11. How easy did you and/or your partner find applying for benefit support? *(eg. DLA, PIP, ESA)*

Very easy ☐ Quite easy ☐ Quite difficult ☐ Difficult ☐ Extremely difficult ☐

12. Did the department dealing with your claim understand your diagnosis?

Yes ☐ No ☐

13. Was your claim successful first time?

Yes ☐ No ☐

14. If no, and you appealed, how many times did you have to appeal until you were successful?

Once ☐ Twice ☐ Three times ☐ I was never successful ☐

15. Did you get what you applied for in the end after appealing?

Yes ☐ No ☐

16. If you are in receipt of benefits do you feel embarrassed by it?

Not at all embarrassed ☐ Slightly embarrassed ☐ Embarrassed ☐ Very embarrassed ☐ Extremely embarrassed ☐

17. If you had to reduce hours or stop working because of PH, were you able to return to work?

Yes, at the same hours as before ☐ Yes, at reduced hours ☐ No ☐

18. If you were employed at the time of your diagnosis, have you ever been given any support from your employer to help you stay or return to work?

Yes ☐ No ☐

Costs related to healthcare

19. ***How many times a month, on average, do you visit a healthcare professional for your PH?***
(include visits to GPs, local and specialist hospitals to see a doctor, nurse, blood tests, heart and breathing tests, imaging etc)

*GP / local hospital*________ *Specialist PH Centre*________ *Other*________

20. ***On average, approximately how much money has it cost you over the last year to visit the healthcare professionals – including travel, parking, any overnight stays*** *(for you or your carer)* ***if needed?***

*GP / local hospital £*__________ *Specialist PH centre £*__________ *Other £*__________

21. ***Since your diagnosis of PH do you spend the same, extra or less on the following items than before?***

	Extra	Same	Less	N/A
Help around the house or garden (eg. cleaner / gardener)	☐	☐	☐	☐
Gas / Electric	☐	☐	☐	☐
General travel costs (eg. to get to shops, see friends, get to work – not including hospital and GP visits)	☐	☐	☐	☐
Household bills (excluding gas and electric)	☐	☐	☐	☐
Childcare	☐	☐	☐	☐
Food costs/ special diet	☐	☐	☐	☐

Insurance costs

22. ***Since your diagnosis, have you applied for life or travel insurance?***
Yes ☐ *No* ☐

23. ***How easy did you and/or your partner find applying for insurances?***
Very easy ☐ *Quite easy* ☐ *Quite difficult* ☐ *Difficult* ☐ *Extremely difficult* ☐

24. ***Since your diagnosis, have you been refused insurance?***
Yes ☐ *No* ☐

25. ***Have insurance quotes been similar / greater/ lower than before diagnosis?***
Similar ☐ *Greater* ☐ *Lower* ☐

General costs

26. ***Since being diagnosed, for financial reasons, have you had to...*** *(please tick all that apply)*

- *Take money from your savings* ☐
- *Cash in part or all of your pension* ☐
- *Borrow from your bank or building society (loan / overdraft)* ☐
- *Take out a credit card* ☐
- *Take out a payday loan* ☐
- *Borrow what you consider a significant amount from your parents* ☐
- *Borrow what you consider a significant amount from your children* ☐
- *Borrow what you consider a significant amount from your friends* ☐
- *Claim on health insurance* ☐
- *Sell some of your belongings to help pay for household bills* ☐
- *Sell your house (for financial reasons caused by your illness)* ☐
- *Visit a food bank* ☐
- *Miss picking up a prescription due to cost* ☐
- *Miss a hospital appointment due to the cost of travel or parking* ☐

27. *Overall, since being diagnosed, do you feel you have more difficulty paying bills?*

No difficulty ☐ *A little difficulty* ☐ *Some difficulty* ☐ *Much difficulty* ☐ *Extreme difficulty* ☐

28. *Overall, after being diagnosed with pulmonary hypertension my financial situation has:*

Improved a lot ☐ *Improved a little* ☐ *Remained the same* ☐ *Declined a little* ☐ *Declined a lot* ☐

29. *Has your condition caused you to fall into debt?*

No ☐ *A little* ☐ *Some* ☐ *A lot* ☐ *Worrisome amount* ☐

30. *If your financial situation has declined, has this affected your mental / emotional wellbeing?*

Not at all ☐ *A little* ☐ *At times* ☐ *A lot* ☐ *Severely affected* ☐

31. *Have you ever accessed or felt the need to access counselling / emotional support* *(eg. cognitive behavioural therapy or similar)* ***as a result of concerns over your financial situation?***

Yes ☐ *No* ☐

If you would like to add any comments relating to how pulmonary hypertension has affected your finances, please do so below (continue on an additional sheet if needed).

Please be assured that the information you provide in this survey will not be shared and will be destroyed once it has been used to input data.

Are you happy for us to contact you in order to clarify any of your responses? Yes ☐ No ☐

If so, please provide your contact details below (there is no obligation to do this).

A THANK YOU FROM US. Would you like to enter our draw for a chance to win one of 150 £10 Love2shop vouchers? Yes ☐ No ☐ *(www.highstreetvouchers.com)*

If so, please provide your contact details below so we can contact all the lucky winners.

Name *Telephone*

Email

Thank you for your time. *By sharing your experiences, you are helping us to support others affected by pulmonary hypertension.*

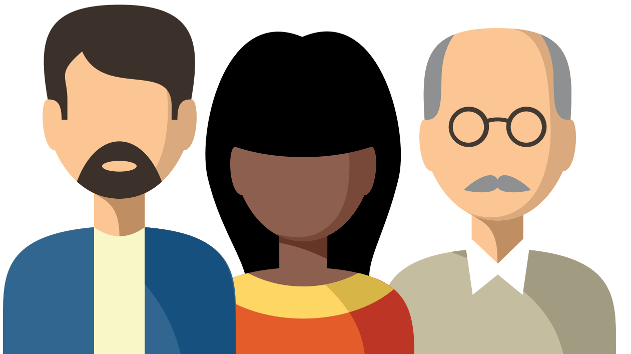

Pulmonary Hypertension Association UK

Tel: 01709 761450 Email: office@phauk.org

Website: www.phauk.org Registered Charity No. 1120756
